# Combined Use of Web-Based and In-Person Education on Ill Health Self-management Skills in Adults With Bipolar Disorder: Protocol for a Mixed Methods Study

**DOI:** 10.2196/25168

**Published:** 2021-09-08

**Authors:** Anna Hatzioannou, Andreas Chatzittofis, Virginia Sunday Koutroubas, Evridiki Papastavrou, Maria Karanikola

**Affiliations:** 1 Nursing Department School of Health Sciences Cyprus University of Technology Limassol Cyprus; 2 Medical School University of Cyprus Nicosia Cyprus

**Keywords:** education, empowerment, bipolar disorders, self-management, bipolar, mental health

## Abstract

**Background:**

Addressing the enhancement of ill health self-management skills in adults diagnosed with bipolar disorder may be considered an important intervention for health care systems worldwide.

**Objective:**

This protocol describes the study “Management of my Bipolarity” (MoB), which aims to develop an educational intervention for adults with bipolar disorder and assess its effectiveness. The objectives include (a) a literature review on bipolar disorder educational interventions; (b) a qualitative exploration of the educational needs of people with bipolar disorder; (c) development of an educational intervention based on objectives (a) and (b) (ie, the MoB educational intervention); and (d) exploration of the effectiveness of the intervention regarding participants’ knowledge of their mental health condition and enhancement of their ill health self-management skills. The MoB educational intervention will consist of an in-person and a web-based intervention in the form of a digital platform.

**Methods:**

The proposed interventional study is a combination of a qualitative and a quantitative design (mixed methods study). A focus group and content analysis will be implemented for the qualitative assessment of the educational needs of adults with bipolar disorder. The intervention will be developed based on the qualitative data of the study and relevant literature. The effectiveness of the acquired knowledge and self-management skills will be assessed according to (a) substance use behavior, (b) health locus of control, (c) impulse control, (d) adherence to pharmacotherapy, (e) relapse prevention, (f) improvement of quality of life, and (g) bipolar disorder knowledge level via structured instruments in the quantitative part of the study using descriptive and inferential statistics (SPSS version 24.0).

**Results:**

A total of 13 patients with bipolar disorder have been interviewed (8 women, 5 men) to identify educational needs to be covered through the intervention. Moreover, a literature review on bipolar disorder educational interventions has been completed. These data have been incorporated in the design of the MoB in-person intervention and the digital platform. The digital platform is live, and the development of the MoB in-person intervention was completed at the end of 2020. The recruitment of the participants for the intervention (40 patients) and the control group (40 patients) began during the first semester of 2021. Moreover, by tracking the platform for 1.5 years, we have recorded that 2180 users have visited the platform with an average session duration of almost 2 minutes. Mobile and tablet devices are being used by 70% of the visitors.

**Conclusions:**

Since new parameters regarding educational interventions will be explored, these findings are expected to provide evidence that participation in structured educational interventions offers patients the opportunity to improve adherence to pharmacotherapy and increase their quality of life.

**Trial Registration:**

ClinicalTrials.gov NCT04643210; https://clinicaltrials.gov/ct2/show/NCT04643210

**International Registered Report Identifier (IRRID):**

DERR1-10.2196/25168

## Introduction

Bipolar disorder (BD) is one of the main causes of disability, affecting approximately 45 million people globally [1,2]. The percentage of people with mood disorders, including BD, in Cyprus is approximately 13.8%, and the incidence per year of people with BD is approximately 0.6% [3]. The most prominent manifestations of BD (ie, cognitive, mood, and interpersonal deficiencies) are all linked with decreased quality of life [4,5]. Therefore, BD has a significant effect on the personal, social, and professional lives of both patients and their families/significant others [6,7]. Most importantly, BD is linked to high mortality rates due to suicide, as well as comorbidities with other types of illness (eg, cardiovascular disease) [8-10]. All of the above highlight the need for support and empowerment of people with BD as one of the key objectives of health care systems worldwide [11].

Empowerment in the context of health care emerges out of the promotion of independence of service users and the restriction of disability and incapacitation, at both personal and social levels [12]. One of the main methods of empowering people with chronic illness is through reinforcement of their capacity to manage the illness themselves (ie, self-management capacity) [12], while reducing health care professionals’ involvement to the minimum possible [13]. Enhancement of ill health self-management capacity is achieved through educational and training interventions [14]. Educational interventions, as therapeutic approaches, include information intake and training in skills regarding the ability to effectively deal with ill health issues such as self-monitoring of symptoms, medication adherence, or fulfillment of daily needs [14,15]. A plethora of positive effects have been associated with enhancement of ill health self-management skills through educational interventions, such as decreased frequency and duration of relapse episodes and hospitalization, and improvement of quality of life [14]. Educational interventions for self-management of disease symptoms have been described for chronic mental diseases, including psychosis and BD [16,17]. Computer software applications have also been incorporated in educational interventions aiming to increase knowledge and health literacy [18,19]. Specifically, web-based interventions present certain advantages such as more opportunities for participation, as they are available throughout the day and provide accessibility to a larger network of people, establishing this method of training as one with a significant financial advantage [20,21]. Moreover, participation in web-based educational interventions may be more favorable for people encountering the social stigma of mental illness, on national and international levels [22,23].

Although previous data support the effectiveness of structured educational interventions for people with BD, the data remain insufficient regarding relevant interventions in northern European countries, including Cyprus, as well as in relation to combined in-person and web-based interventions [14]. Specifically, there are no relevant structured interventions available in the Greek language. In addition, to the best of our knowledge, only one study reported to date used a combination of in-person training alongside a respective web-based intervention [24]. Although numerous digital educational interventions and relevant platforms for BD exist, the degree to which they have been developed according to any theoretical background or empirical data on the needs of patients is not clear. Notably, no digital platforms aiming to increase the knowledge and educate people in BD or enhance relevant ill health self-management skills have been found in the Greek language, and the implementation of such interventions appears to be almost absent in the health care systems of Greece and Cyprus.

Previous data support difficulty in impulse control [25], and high rates of substance use [26] and nonadherence to pharmacotherapy [27] in people diagnosed with BD, which are all linked to increased relapse rates and decreased quality of life [4,5]. Thus, interventions, mainly psychotherapeutic, toward addressing these issues need to be further explored [28]; the combination of in-person and web-based interventions is highly recommended for effective management of symptoms [29]. Other studies support that performance of ill health self-management skills is associated with one’s self-perception about the health-related locus of control and related health literacy level [30,31]. Overall, educational interventions targeted to people with chronic disorders, including BD, should constitute a basic service offered by health care systems and mental health service structures at the international level [11].

The proposed protocol focuses on a study aiming to develop an educational intervention for patients with BD, and explores the null hypothesis that patients who solely receive pharmacotherapy will show the same improvement in important aspects of their lives, such as relapse rate and quality of life, compared to those who receive pharmacotherapy and structured educational interventions. The effectiveness of the proposed intervention will be tested in relation to improvement with respect to (a) impulse control, (b) substance use attitudes, (c) adherence to pharmacotherapy, (d) relapse prevention, (e) BD knowledge level, and (f) self-perceived quality of life and locus of health-related control. Thus, the findings of the proposed study are expected to provide data on the effectiveness of a structured educational intervention, which combines in-person and digital interventions, regarding enhancement of ill health self-management skills.

## Methods

### Aim

The aim of the study entitled “Management of my Bipolarity,” hereafter referred to as MoB, is to develop and test an educational intervention for patients with BD. The stages of the study include: (1) a literature review on BD educational interventions; (2) exploration of the educational needs of adults with BD; (3) development of the MoB educational intervention, encompassing in-person sessions and a digital platform; and (4) exploration of the effectiveness of the MoB intervention in adults diagnosed with BD regarding (i) their knowledge about this disorder, and (ii) the empowerment of ill health self-management skills in relation to the improvement of impulse control, adherence to pharmacotherapy, relapse frequency, quality of life, and substance use attitudes.

### Design

This study is based on a mixed method design and consists of four stages.

Stage 1 includes a qualitative exploration of the educational needs of patients, aiming to develop an experimental educational intervention according to these needs. Moreover, a literature review on BD educational interventions was performed in this stage to support this objective according to the steps described by Tawfik et al [32] for systematic reviews. Stage 1 has been completed.

Stage 2 includes the design of the MoB intervention both in person and with the digital platform. The design of the in-person intervention relies on the Colom and Vieta [33] model, in which cognitive behavioral techniques are incorporated according to existing literature [34] and the results of Stage 1 (all relevant findings of the literature review and data acquired in the qualitative research of educational needs). The structure of the MoB digital platform has been designed by the researchers of this study and a web developer, partially based on the preferences and needs of the participants (as determined in Stage 1). Stage 2 has also been completed. The digital platform will be further enriched based on the qualitative and quantitative findings from platform evaluation techniques and interactive educational features in Stage 4.

Stage 3 includes implementation of the MoB in-person educational intervention and the quantitative evaluation of its effectiveness regarding the acquired knowledge and self-management skills of the participants at four time points (ongoing).

Stage 4 includes the qualitative and quantitative assessment of the applicability of the digital platform (ongoing).

### Settings and Sampling

The target population is adults diagnosed with BD who are under treatment. The recruitment will be implemented in collaboration with the mental health services used by the participants. Specifically, both private and public mental health services in Cyprus have been informed about the objectives and procedures of the study through a specific communication process. Through this process, several patients have already been referred to the research team by their psychiatrists/therapeutic team coordinator to participate in Stage 1. As a result, convenience sampling will be combined with snowball sampling, according to study inclusion criteria set in every stage. The study is also posted on social media and groups related to BD (health care service users, health care professionals).

To promote participant retention, an action plan has been developed. Specifically, the principal investigator (AH) will arrange one or two social meetings with the participants of Stage 1 and Stage 3 to achieve some degree of intimacy, emphasize confidentiality, and ensure engagement with the study. The cost of these social meetings is covered by the Nursing Department of Cyprus University of Technology. Regarding the control group of Stage 3, the principal investigator (AH) will also make a phone call once or twice per month to reassure their participation in the study, as follows: “Hello, I am Anna from the MoB project. I’m calling to see how you are, and if you are still interested in participating in the project. The next appointment will be on date X. Is this okay with you, or do you have any other arrangements for the day?”

### Stage 1

#### Objectives

The objective of Stage 1 is to perform a literature review on BP educational interventions and define the educational needs of adults with BD via empirical exploration of their living experience, aiming to integrate data in the MoB intervention. This stage has been completed. Specifically, the objectives of the systematic review included exploration of the educational methods applied to individuals with BD and their effectiveness regarding enhancement of ill-health self-management skills in relation to (i) relapse prevention, (ii) adherence to pharmacotherapy, and (iii) enhanced quality of life. This stage also evaluated the BD literacy needs in people with BD. An additional objective of the review was to inform the design of Stage 3 regarding eligible outcome measures for assessment of the effectiveness of the proposed intervention.

An advanced search in the CINAHL Medline, Scopus, Psych INFO, and Cochrane Library databases was performed between December 2018 and June 2020 by two researchers (MK and AH). The following key words were used singly and in combination: “bipolar disorder,” “manic-depress*,” “mania,” “depress*,” “education*,” “self-management,” “intervention,” “program*,” “empowerment,” “psycho-education,” “e-health,” and “literacy.” The following inclusion criteria were set: (a) an empirical, quantitative study; and (b) published in the English language in a peer-reviewed journal between 2007 and 2020. The methodological accuracy of the included studies was assessed with the Health Evidence Quality Assessment Tool [35].

#### Inclusion Criteria and Sampling for Qualitative Exploration of Patients’ Educational Needs

The inclusion criteria were set as follows: (a) sufficient experience (more than 6 months) as a patient under BD treatment; (b) willingness to communicate the living experience of BD; and (c) ability to reflect on the living experience of BD, as it will be drawn from the narrative content [36]. The final sample size was based on theoretical and data saturation of emergent themes [37-39].

#### Data Collection and Analysis

The form of the data collection interview, focus group or personal interview, was designed to be assigned according to the participant’s convenience. Since Cyprus is a small island with approximately 1 million citizens, some of the participants were reluctant to participate in focus group discussions due to the social stigma of mental illness [40]. Participation in personal interviews was expected to preserve their anonymity.

A semistructured guide with open-ended questions, set up by the research team according to relevant literature, was used for data collection in this stage. The interview guide included the following questions:

1. Please tell us what you know about your mental health condition/BD.
2. Please describe what you would like to know about bipolar disorders.
3. Please describe in what way and on what topics you would like to be educated and increase your knowledge on bipolar disorders?
4. Please describe your expectations from an educational intervention regarding its impact on your everyday living.
5. Would you like this intervention to be implemented one-on-one or in a group?
6. How long would you like the intervention to last?
7. What is your level of engagement with the internet?
8. Are you familiar with computer use?

The original intention was to have two focus groups (6-10 people per group); however, in the process of the research, only one focus group was ultimately deemed to be necessary. The focus group participants met two to three times. In the second meeting, the themes revealed in the first meeting were verified and enriched where possible. During the third meeting, the researchers interpreted the data, followed by a discussion with the participants. This method was also applied to personal interviews (2-3 meetings with each participant). Data analysis was based on the conventional mode of content analysis [41].

### Stage 2

The purpose of the development of the ΜoΒ in-person intervention is to create an applicable, feasible, and effective intervention to enhance BD knowledge and ill-health self-management skills in people diagnosed with BD. Specifically, a textbook has been devised explaining in detail the step-by-step process and the techniques of the experimental educational method of the MoB in-person intervention.

The goal of the development of the MoB digital platform is to create an ecosystem where people with BD will be educated and empowered to make use of the platform via a computer, tablet, or mobile phone. The owner of the platform is the Nursing Department of Cyprus University of Technology and the administrator is the principal investigator (AH). The participants of the proposed study will have the opportunity to access the platform during and after completion of the study.

### Stage 3

#### Inclusion and Exclusion Criteria

The following inclusion criteria have been set for participation in the MoB intervention: (1) clinical diagnosis based on the Diagnostic and Statistical Manual of Mental Disorders-5 for BD; (2) aged between 18 and 65 years; (3) signed the informed consent form; (4) stable mood status at the beginning of the MoB in-person intervention based on clinical assessment, conducted by the principal investigator (AH); (5) experience of the illness for at least 1 year based on the medical record; (6) adequate awareness of the illness, based on the following guide: (a) are you aware of the reason(s) you are using mental health services/under medication? and (b) are you aware of the aim of the educational intervention in which you may participate?; and (7) familiarity with computer use.

The exclusion criteria are: (1) intellectual disability (IQ<70), based on the Wechsler Adult Intelligence Scale [42]; (2) brain damage (eg, following a stroke) based on diagnostic tests; (3) acute phase of the illness, based on clinical assessment and use of the Young Mania Rating Scale (YMRS; total score <12) [43] and/or Beck Depression Inventory (total score <17) [44]; and (4) substance use problems at the beginning of this stage according to the Alcohol Use Disorders Identification Test (AUDIT; total score <9) [45] and Drug Use Disorders Identification Test (DUDIT; total score <2 for women or <6 for men) [46].

All eligible participants will be randomly assigned (in terms of gender, age, duration of illness) into the intervention group and the control (waiting) group. With the aim of obtaining a moderate correlation effect, with 80% statistical power and .05 level of statistical significance, 40 participants are needed in the intervention group and 40 participants are needed in the control (waiting) group.

The criteria for discontinuing participation in the intervention group are disorder relapse and nonadherence to pharmacotherapy (discontinuation of medication).

#### Intervention Procedure

The form of this intervention will be in groups or in person according to the participant’s choice (similar to the data collection process as detailed above). Additionally, all participants of this group will have access to the MoB digital platform and will be assessed via a checklist on the frequency and extent to which they use the digital platform in each in-person session.

The implementation methodology of the MoB in-person intervention will include videos and PowerPoint presentations, as well as interactive learning methods such as role playing, empowerment exercises, and live discussions.

The MoB in-person intervention will comprise a total of 12 educational sessions. Each session will have a duration of 1.5 hours and the maximum number of participants in the groups will be 12 patients. At the beginning of each session, the participants will be given the opportunity to record their feedback for the previous session and the impact of the training intervention so far for themselves and for their families/significant others. The intervention will be performed at Cyprus University of Technology and will be implemented by the principal investigator (AH). Participation in the educational intervention is free of charge. Moreover, there is no financial benefit for those participating in the study (participants, researchers) in terms of reimbursement or any other material benefit.

Those who wish to withdraw participation prior to completion of the intervention will be able to continue to receive information/knowledge about their illness through the digital platform.

The control (waiting) group will only have access to the MoB digital platform. Yet, it should be noted that the control (waiting) group will also receive the MoB in-person intervention if its effectiveness is shown at the end of the study. In this way, all participants will be given the chance to receive the MoB intervention.

#### Effectiveness Evaluation

Evaluation of the effectiveness of the MoB in-person intervention will be based on the degree of improvement in the following outcome variables defined according to the findings of the literature review completed in Stage 1: (1) self-perceived quality of life level, as measured by the World Health Organization Quality of Life Assessment (WHOQOL) tool [47]; (2) self-perceived health-related locus of control, as measured by the Multidimensional Health Locus of Control (MHLC) scale [48]; (3) ability to control impulses, as reflected in one’s sexual and aggressive behavior, measured by the items “aggressive behavior” and “sexual interest” of the YMRS [43]; (4) substance use problems, measured by the AUDIT (alcohol use) [45] and DUDIT (drug use) [46] tools; (5) adherence to pharmacotherapy, measured by the Drug Attitude Inventory (DAI-30) tool [49]; (6) frequency of relapses assessed by the reduction of the number and duration of hospitalizations for the 2 years following the end of the educational intervention; and (7) BD knowledge level assessed by the Bipolar Disorder Knowledge Scale (BDKS) [50].

The degree of self-perceived quality of life will be measured by the total score in the WHOQOL scale assessment. This is a 1-5 Likert-type scale including 26 items. The minimum score in the scale is 26 and the maximum is 130; higher scores indicate a better outcome. The BD level of knowledge will be measured by the total score in the BDKS. This is a 25-item, true-false scale. The items address diagnosis, etiology, disease course, symptoms, treatment, and life impact BD-related knowledge. The ratio of “true” to “false” responses is assessed; a higher percentage of correct values indicates a better outcome. The degree of ability to control impulses will be measured by the total score in the YMRS. This is a 0-4 Likert scale, including 11 items. The total score of the scale ranges from 0 to 44; a lower total score indicates a better outcome. The degree of adherence to pharmacotherapy will be measured by the total score in the DAI-30. This is a 30-item true-false scale. The ratio of “true” to “false” responses is assessed; a higher percentage of correct values indicates a better outcome. Drug use will be measured by the total score in the DUDIT scale. This is a 1-5 Likert, 11-item scale (total score range: 1-55); a lower total score indicates a better outcome. Alcohol use will be measured by the total score in the AUDIT. This is a 0-4 Likert, 10-item scale (total score range: 0-40); lower scores indicate a better outcome. The degree of self-perceived health-related locus of control will be assessed by the MHLC tool, which is a 6-point Likert (scored from 1 to 6), 18-item scale (Form A). These items are grouped in three subscales corresponding to three different loci of control (ie, internal, others, and fate/luck); higher sum scores in each of the 3 subscales indicate a stronger locus of control. The frequency of relapses will be measured by the number of hospitalizations after completion of the in-person intervention. The number of hospitalizations will be measured as the sum of individual hospitalizations in a high-security psychiatric hospital/setting; the lower the number of hospitalizations, the better the outcome. The duration of relapses will be assessed by the duration of inpatient hospitalizations in days after completion of the in-person intervention. The duration of inpatient hospitalizations will be measured as the sum of the days of hospitalization in a high-security psychiatric hospital/setting; the lower the duration in days of hospitalization, the better the outcome.

There will be four evaluation time points. The first evaluation time point will be prior to implementation of the MoB in-person intervention, the second will take place immediately after completion of the MoB in-person intervention, and the third and fourth evaluations will be applied at 6 and 12 months, respectively, after completion of the MoB in-person intervention. These time points have been determined according to the systematic review completed in Stage 1 [14].

The control group will be assessed via the same evaluation tools as the intervention group, and at the same time periods. This will test the null hypothesis that patients who solely receive pharmacotherapy demonstrate the same improvement as the intervention group.

The educational meetings will be held at Cyprus University of Technology in a room specially designed for this purpose that is soundproof, without windows, and chairs placed in a circular arrangement to allow for eye contact, along with small tables on either side to keep notes.

#### Data Analysis

The means (SD) will be calculated for the scale numeric (sum) scores (YMRS, WHOQOL, MHLC, BDKS, DUDIT, DAI-30, AUDIT, relapse frequency/duration) and frequencies will be calculated for categorical variables (gender, age groups, sex, years in illness groups). Parametric tests (analysis of variance, *t* test) will be applied for comparisons between groups. For all statistical tests, *P* values of .05 or lower will be considered statistically significant. Data will be analyzed through the Statistical Package for Social Sciences (SPSS Inc, version 25.00). To test the relationship between two or more variables, logistic and linear regression analyses will be used. The multiple imputation method will be applied to handle missing data.

### Stage 4

Assessment of the applicability of the MoB digital platform will be based on qualitative (users’ feedback) and quantitative (data analysis) testing. The qualitative assessment will be based on the experience of the users with the platform and on the degree to which they were positively affected by its use. Specifically, the focus of the interviews will be on the impact of the platform on their everyday life, as well as on enhancement of ill health self-management skills according to the participants’ responses to the following open-ended questions: (a) “How would you describe your experience with the use of the MoB digital platform?” (b) “What was the impact of use of the MoB digital platform on your life?” and (c) “What was the impact of the MoB digital platform on your ill health self-management skills?” This process will take place via focus groups discussions. Specifically, two focus groups with 8-12 participants each will be recruited including participants who (a) attended the MoB in-person intervention and had used the platform and (b) solely used the MoB digital platform.

The quantitative testing involves anonymous questionnaires provided to users of the MoB digital platform, who will have to answer predefined questions regarding satisfaction parameters relevant to the platform. The first outcome assessed will be the level of users’ satisfaction with the MoB digital platform, measured by the score on a 3-item Likert scale (low, moderate/accepted, high) self-questionnaire exploring users’ perceptions of overall experience; utility, according to their present medical condition and clinical state; practicality on daily usage; improvement of self-management skills; and technical difficulties experienced [51]. The second outcome is the degree of improvement in users’ knowledge as measured by the score on the BDKS [50].

### Patient and Public Involvement

Although patients with BD have not been included as members of the present research team, their input in the development of this study will be constant and accounted for during various stages of this project. Patient/participant involvement is registered as follows. Once enrolled in the study, participants’ feedback and reactions will be collected by the research team and used for improvement of the implementation of the study protocol. Respect for the rights, experiences, and personalities of the patients involved is one of the key priorities and motivations of the research team. During stage 1, the qualitative (empirical) exploration of the educational needs of patients with BD will be assessed. During the development of the MoB in-person intervention, the features, methods, and stages described by the participants (data from Stage 1) will be taken into account. Participant perspectives will also be addressed during preparation of the research questions on the effectiveness of the MoB in-person intervention. These questions are expected to be finalized according to the preliminary input resulting from the participants’ narratives on the clinical areas that need to be addressed by the MoB in-person intervention and the expected impact of the intervention on their lives (data from Stage 1). The issue of the participants’ burden of the MoB in-person intervention is included in the interview guide. Relevant data were used to define the duration of the MoB in-person intervention and the assessment periods of the intervention (data from Stage 1). Τhe participants are expected to introduce the proposed study to their peers, since a snowball sampling technique will be applied in Stage 3 aiming to recruit the participants of the intervention and control group. Finally, the participants who will be involved in Stage 4 will assess the applicability of the MoB digital platform via qualitative and quantitative data.

Due to the limited population of the Republic of Cyprus, attracting an adequate number of participants for the study is one of the challenges faced. However, the research team will disseminate the call to patients and providers’ associations as well as through other available means to ensure patient participation.

Members of the public, or the significant others/family members of the participants, will not be involved in the study. However, should the intervention be considered successful, significant effort and work will be exerted to alleviate the effects of the stigma of BD in society, both in Cyprus and internationally. A collaborative effort will be undertaken by the researchers to engage with members of the public to highlight the experience of BD, and the significance and effectiveness of the MoB intervention.

### Registration and Ethics

This protocol was approved by the Cyprus National Committee of Bioethics (ΕΕΒΚ/ΕΠ/2018/27) on September 30, 2018, and was registered at the Research Committee Review board of the Ministry of Health of the Republic of Cyprus (5.34.01.7.6^Ε^/0490/2018) on February 18, 2019. Moreover, the research protocol has been registered in ClinicalTrials.gov (ID: NCT04643210). All participants have/will sign the consent form for participation in the study, which includes the parameter of data publication. The questionnaires included in this study protocol have been previously published elsewhere and all relevant references to the questionnaires are cited in the manuscript.

## Results

### Stage 1

#### Literature Review

The literature review has been completed, and these data have been published [14]. Specifically, 15 studies were included in the review, showing that effective management of BD was mainly based on the combination of pharmacotherapy and structured educational interventions. Participation in structured educational interventions was associated with improvement in global functionality, adherence to pharmacotherapy, and early detection of relapse symptoms, all resulting in reduced frequency of hospitalization and experiences of mental illness social stigma. However, none of the reviewed studies assessed important areas of functioning (ie, cognition, delinquency, and impulse control). In relation to the mode of the educational intervention, both digital and in-person interventions were identified. Regarding the structure of the reviewed educational interventions, the majority were developed according to modified versions of the Colom and Vieta model [33], and their duration ranged with 7-8 in-person sessions and 6-21 group sessions. Moreover, personal interventions were linked to an increased frequency of drop-off and decreased duration of positive effects compared with those of group-based interventions. However, additional research on the effectiveness of in-person interventions compared to group-based interventions was suggested. Although this review described evidence on the effectiveness of web-based educational interventions, relevant data were not sufficient or of adequate quality to further support their usefulness. Nevertheless, it was revealed that web-based interventions were associated with advantages such as availability throughout the day and subsequently more opportunities for participation, accessibility to a larger network of people, and cost-effectiveness. Moreover, web-based interventions were more attractive to those encountering social stigma and younger participants. Additionally, the vast majority of the studies reported on the increased patient satisfaction from participation in educational interventions, and the most frequent reasons for quitting these interventions were increased job demands, relapse, and lack of time. Moreover, those with a duration of illness of more than 15 years did not benefit significantly from educational interventions. In conclusion, this review suggests the need for further intervention studies in educating BD clinical groups, combining in-person and web-based interventions, and further assessment of their effectiveness in areas such as substance use, delinquency, and impulse control.

#### Empirical Qualitative Exploration of Educational Needs

Regarding the qualitative exploration of the educational needs of ill-health self-management skills in people diagnosed with BD, a total of 13 participants were interviewed (8 women, 5 men). Specifically, since participation in interviews was designed to include both personal and focus group modes, we completed one focus group with 6 participants and 7 personal interviews (May to September 2020). The simultaneous data collection and analysis of emerged themes allowed us to determine thematic saturation up to this sample size. Thus, we did not proceed to an additional focus group as intended. Moreover, the fact that the participants came from different educational, social, and professional backgrounds supported the criterion of data saturation. By April 2021, all three meetings had been completed with all 13 participants. Relevant data defined the design of the MoB intervention and are expected to be published by the end of 2021.

Specifically, the participants only partially described the main symptomatology and the risks stemming for inadequate management of BD, and subsequently the impact of BD on their lives; notably, none of the participants reported any type of participation in a structured educational intervention on BD. Furthermore, the internet was identified as the primary source of education about their illness, with health care professionals (ie, psychiatrists and mental health nurses) listed as the second, yet ineffective, source of knowledge. Regarding BD-related educational needs, the participants mainly focused on the necessity to become familiar with the pathophysiology and symptomatology of BD, but most importantly the etiology behind the onset of this disorder and the factors that might have triggered BD. Moreover, they clearly described their need to enhance their knowledge about pharmacotherapy, side effects, and the rationale behind particular types of psychotropic agents, as well as effective management of the adverse effects of pharmacotherapy. Substance use was also included in the agenda of the participants regarding their educational needs. Additionally, the participants reported their need to be educated on how to manage human/patient rights violations against them, mainly in relation to consent to treatment, participation in clinical decision-making, and capacity to make legal decisions. The participants also described their need to enhance knowledge about the therapeutic procedures applied in other countries, as well as in relation to complementary therapeutic options. Female participants expressed their need to learn more about the link between BD and reproductive and sex-life health issues, with focus on fertility and the link between pharmacotherapy side effects and menstrual disturbances, childbirth, and hormones regulation. Employment-related issues were also raised, especially with respect to lack of knowledge regarding sick-leave issues and disclosure issues about their health status to their employers and coworkers. The participants also provided input regarding the frequency, duration, and mode of educational sessions. Most importantly, the participants underlined their need to be practically educated on how to implement all of the provided information into their daily lives, and mainly on how to identify and self-manage (if possible) early symptoms to prevent relapse.

Regarding the above topics, those referring to (a) sexual and reproductive health, (b) patient/human rights, (c) employment issues, and (d) alternative and complementary therapeutic options were incorporated into the MoB intervention, since these topics were not included or adequately covered in the Colom and Vieta [33] module. Moreover, the participants’ suggestions about the frequency (once a week), duration (90 minutes per session/12 sessions), and mode (both personal and group-oriented options to participate in the MoB intervention, combination of in-person and online modules via a digital platform) were also taken into consideration during development of the MoB intervention.

Beyond educational needs, the participants spontaneously provided descriptions about social needs, and specifically expressed their need to belong to a certain group or society, as well as their willingness to support others in need. Based on this input, a session on social skills training and information about how to engage in volunteering was incorporated in the design of the MoB intervention.

### Stage 2

#### MoB In-Person Intervention

A textbook explaining the detailed, step-by-step process and the techniques of the experimental educational method of the MoB in-person education has been devised. According to the data acquired in Stage 1, the MoB in-person intervention comprises a total of 12 in-person educational sessions, with one session per week. Each session has a duration of 1.5 hours and the maximum number of participants in the groups is 12 patients.

#### MoB Digital Platform

The interactive part of the platform is under development, while the informative part is live [52] and accessible to the participants in the current study, as well as to any interested person (eg, family members of people with BP) or caregivers providing updated information related to BD (platform users). The platform is in the form of a dynamic website with user-generated content as well as static information, following a responsive design (ie, accessible with all types of devices). The informative part, so far, includes the following functionalities: self-management action plan, homework exercises, self-assessment tools, and scientific articles relevant to BD. Entry into the platform functionalities (scientific articles excluded) is password-protected.

Users can register in the platform by filling in their personal profile and can log out/log in and revisit at their convenience. Regarding self-management functionalities, the platform will contain subject units where personal relapse symptoms may be registered, a life events table, personal risk factors for relapse, and a weekly program of activities. Input of relevant information will be provided through open-ended questions and checklists. Participants of the MoB in-person intervention who visit the platform will find that these subject units (chapters) are topics on which they will be educated on. Additional features will be incorporated according to development of Stage 1 (eg, new training needs as described by participants in the focus groups).

Another subject unit will be the personal therapy file, where users will have the chance to keep tabulated notes related to their therapy, including information about the dose and type of medication treatment, as well as pending medical tests (eg, blood tests) and other relevant information. The therapeutic team of the user will have the ability to access the information by registering in the platform under approval of the user with the aim to support continuity and share information.

### Stage 3

The recruitment of the participants of the intervention group (40 patients) and the control group (40 patients) began during the first semester of 2021.

### Stage 4

User behavior data have been analyzed through Google Analytics by tracking the platform for approximately 1.5 years (January 2019 to October 2020). Specifically, we have recorded that 2180 users have visited the platform with an average session duration of almost 2 minutes. The bounce rate of these visits is 68%, which calls for improvement. The returning visitor rate is approximately 10%, and 70% of the visitors are accessing the platform via mobile and tablet devices.

The website has a total of 7100 page views. Each user has an average of 2.6 pages per visit. In terms of search appearance, it seems that 72% of the traffic is derived from organic searches, which means that search engine optimization fundamentals have been applied to the highest level. Usability testing is being performed to ensure that the website is improving the lives of the users.

The full development and release of the interactive part of the digital platform is expected to be finalized by the second semester of 2022, at which time the assessment of the applicability of the platform will take place.

**Figure 1:**
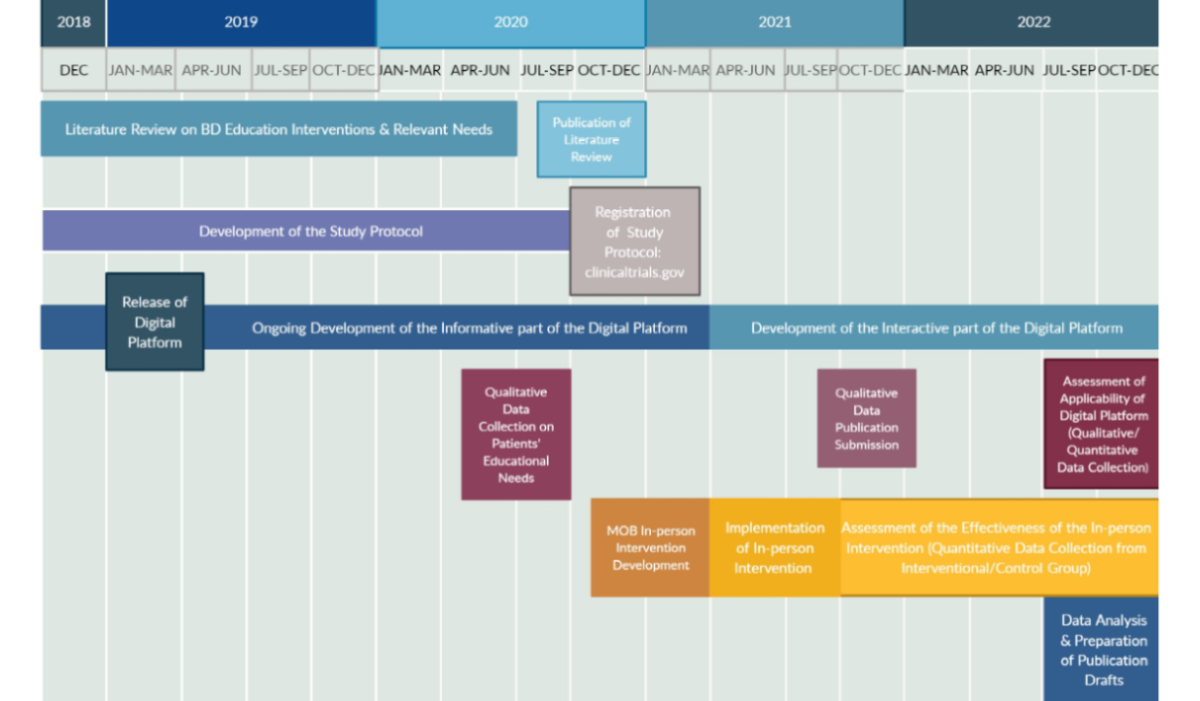
Timeline of the Management of my Bipolarity (MoB) study. BD: bipolar disorder.

The timeline of the proposed study is presented in Figure 1.

### Confidentiality and Dissemination

#### Ethics and Data Management

Participation in all stages of the study will follow the provision of informed consent. The participants will be informed both orally and in written form about the purpose and process of the study, as well as about confidentiality issues regarding the revealed experiences and the safe storage of the collected data. The principal investigator (AH) will answer all questions. The consent form will include a telephone number and email address for expressing complaints relating to the procedures of the study. Participants will be able to withdraw at any point as they wish, with no repercussions in the therapeutic procedure they already follow.

Each interview will be recorded with the participant’s permission. Moreover, the participants will be reassured that no data that could reveal their real identity would be presented or reported at any point of the study. Pseudonyms will be used for participation in the study. Although there is no identified psychosocial or other type of danger or harm related to participation in this study, the research team will provide psychosocial supportive services to those in need during the study and for up to 1 year after its completion.

#### Dissemination Policy

Dissemination of the results will take place through publications in international and national scientific journals. There will also be presentations of the data in nursing and medical conferences at national and international levels. All publications and presentations that will be held will include the names of all the collaborators who contributed to the implementation of this study. The aim of the dissemination is to inform health professionals and researchers interested in the follow-up of similar methodological planning for intervention studies.

#### Data Management

The data collected from each participant will be encoded as a letter according to the group to which they will be assigned (ie, “I” for the intervention group or “C” for the control group) and the sequence number of entering this group (eg, I1 or C1). This algorithm is expected to produce a unique code for each participant. All data will be safely stored in a locked cabinet in the office of the main supervisor, MK. Access is granted only to MK and AH. The data will also be stored on the computer of the main supervisor. When the data are stored on the computer, AH and MK will perform a double check to assure that the questionnaires have been completed accurately. The data will be published immediately in scientific publications for their verification. Mechanisms to protect data, based on the General Data Protection Regulation, have been taken into account to ensure the security of sensitive information provided on the digital platform by its users. In relation to security of data linked to any kind of communication within the environment of the platform, the HTTPS encryption system will be applied. Moreover, in relation to privacy issues, a cookies system will be applied. Access to personal files will be password-protected and all relevant information will be encrypted. Identification issues will also be taken into consideration. Overall, further investigation on security, privacy, and identification issues will be ongoing.

## Discussion

### Projected Findings and Significance

The findings of the proposed protocol will shed some light on the characteristics of structured educational interventions applied to people with BD and the educational needs of this clinical population. Most importantly, the proposed study is expected to provide data on the effectiveness of a structured educational intervention regarding enhancement of ill health self-management skills in this clinical population. The development of a relevant digital platform is also expected to contribute to this goal. Structured educational interventions include knowledge provision and experiential exercises to adapt effective attitudes regarding the self-management of BD [20,53-66], while the degree of self-management of chronic mental disorders is further linked with improved patient quality of life [67,68].

The review performed in Stage 1 of this protocol revealed that control of the symptoms of BD is a complex process, which appears to be based on the combination of pharmacotherapy, psychotherapy, and structured education [20,53-66]. Moreover, this review confirmed that participation of people with BD in structured educational interventions gives them the opportunity to improve important life parameters such as functionality in terms of fulfillment of everyday life activities and work engagement [16], adherence to pharmacotherapy, early detection of relapse symptoms, reduction in the frequency and length of hospitalization [69], and limitation of social stigma [65]. Additionally, it became clear that individuals with BD who participate in training interventions have fewer symptoms of intensity, resulting in a decrease in the amount of medication received [70]. Moreover, based on this review, it was shown that the duration of relevant educational interventions varies according to the form of the training intervention (individual- or group-oriented). Specifically, 6 to 21 training sessions seem to be necessary for group interventions and 7-8 sessions are needed for individual interventions [16,54,60]. Another factor found to be related to the effectiveness of education was the chronicity of the disorder. Those who described a chronic state of the disorder with a duration of more than 15 years did not benefit significantly from educational interventions [47]. Subsequently, priority should be given to educational interventions in the early stages of diagnosis of the disorder to achieve the highest possible efficacy. On this basis, since both the intervention and control groups in the proposed study will include individuals with a variety of illness durations, caution will be taken during data analysis to control this confounder.

Integration of the qualitative data of Stage 1 into the development of the MoB intervention was achieved in relation to both the curriculum of the intervention and the delivery mode. Most importantly, since the participants confirmed a lack of structured, culturally adapted educational interventions in the Greek language, we may assume the necessity of applying the proposed educational intervention to people diagnosed with BD and their families in an accessible and friendly mode; thus, implementation of advanced, web-based educational interventions accessible to both clinical and nonclinical populations may be supported. Nevertheless, the internet was identified as the primary source of education for the participants, which was also described as a useful source of knowledge. Consequently, the development of a culturally sensitive platform providing reliable and updated information and knowledge to the public may be deemed necessary. Overall, based on the above, the research team confirmed the need to combine in-person sessions with a digital platform in the form of a dynamic website with user-generated content as well as static information, while these data have been integrated into the design and assessment of the applicability of the MoB digital platform.

Regarding the context of the MoB educational sessions, several new topics were introduced: (a) sexual and reproductive health, beyond lithium-related topics and sexual impulse control in mania/hypomania, which are already included in the Colum and Vieta [33] model; (b) patient/human rights; (c) employment issues; and (d) alternative and complementary therapeutic options, beyond the stress-control techniques already included in the Colum and Vieta [33] model. Additionally, aiming to support the participants’ social needs, a session on social skills training and information on how to engage in volunteering was incorporated into the MoB intervention.

Based on the preliminary metrics related to the digital platform, despite low rates of engagement, bearing in mind the rate of visitations, we may conclude that there is an interest in the digital platform. The numbers of visitations may further suggest that we have developed a user-friendly platform following the latest technological trends. Nevertheless, it would be useful to know how many visitors typically visit other similar websites regarding patient education, and to further compare the rate of visitations of the digital platform with the typical rates for such resources. Currently, due to a lack of websites in the Greek language on psychiatric patient education, a relevant comparison was not possible. Overall, further improvements will be made based on ongoing data retrieval and testing, with special focus on ways to increase the duration of engagement in the platform. The qualitative and quantitative assessment of the platform is still ongoing, which is expected to be finalized at the end of 2021.

Approximately 5.7 million adults in the United States are diagnosed with BD annually, a percentage corresponding to 2.6% of the total adult population [70,71], while BD is the second most prevalent neurobiological (mental) disorder in the Republic of Cyprus, following psychotic thought disorder [71]. The median age of onset for BD is 25 years, although the illness can start in early childhood or late adulthood, having a long-term impact on one’s life [72]. An equal number of men and women develop BD, and this condition is found in all ethnic groups and social classes [72]. Most importantly, BD results in a 9.2-year reduction in expected lifespan, and as many as 1 in 5 individuals with BD attempts suicide [72]. Additionally, a prolonged lack of diagnosis or false diagnosis at some point of the illness trajectory is a common phenomenon among people with BD [73]. As a result of delayed effective treatment, the symptoms of the disorder become more severe, along with an increased frequency of relapse [74,75]. All of the above constitute the application of innovative, multidimensional, and effective therapeutic approaches aiming to increase the quality of life of this clinical group and adequately empower patients as an imperative goal. Empowerment in the context of health care refers to the status in which service users may function under the highest degree of autonomy, achieve their personal life goals, and gain the optimum level of quality of life [76,77]. Studies on the effectiveness of therapeutic methods aiming to empower ill health self-management skills in people with chronic illness, including BD, have been reported to be necessary [78,79].

Several changes have been made to mental health services in Cyprus; today, the majority of services are provided in community rather than institutional settings. According to the goals of the Ministry of Health of the Republic of Cyprus, advanced, evidence-based mental health clinical practices are considered an integral part of the therapeutic interprofessional approach toward clinical populations [71]. However, to our knowledge, there is no evidence of the extent to which empowering-oriented approaches toward individuals diagnosed with BD have been incorporated to the recent modification of mental health services. Implementation of the proposed intervention is expected to contribute to this goal in Cyprus.

### Limitations

Regarding the limitations of the proposed study, only adults will be involved (18-65 years), which will limit the ability to obtain a comprehensive depiction of the effectiveness of the MoB intervention in populations of different ages (eg, adolescents, the elderly). Additionally, the interventions will be developed only in the Greek language, excluding non-Greek–speaking participants, leading to limited generalizability of the findings.

### Conclusion

This is the first study, to the best of our knowledge, on structured education of adults with BD that combines in-person and web-based interventions. The outcome of robust evidence on the effectiveness of the MoB educational intervention is expected to contribute to the development of knowledge and documentation on the subject, locally in the Greek-Cypriot area, but internationally as well. New parameters regarding educational interventions will be explored, while data from the proposed study are expected to support the incorporation of the MoB educational intervention into mental health care services, thereby enhancing mental health nursing practice, both in Cyprus and globally.

## Abbreviations

AUDIT: Alcohol Use Disorders Identification Test
BD: bipolar disorder
BDKS: Bipolar Disorder Knowledge Scale
DAI-30: Drug Attitude Inventory
DUDIT: Drug Use Disorders Identification Test
MHLC: Multidimensional Health Locus of Control
MoB: Management of my Bipolarity
WHOQOL: World Health Organization Quality of Life Assessment B
YMRS: Young Mania Rating Scale
