# Correction: Combined Use of Web-Based and In-Person Education on Ill Health Self-management Skills in Adults With Bipolar Disorder: Protocol for a Mixed Methods Study

**DOI:** 10.2196/33506

**Published:** 2021-09-15

**Authors:** Anna Hatzioannou, Andreas Chatzittofis, Virginia Sunday Koutroubas, Evridiki Papastavrou, Maria Karanikola

**Affiliations:** 1 Nursing Department School of Health Sciences Cyprus University of Technology Limassol Cyprus; 2 Medical School University of Cyprus Nicosia Cyprus

In “Combined Use of Web-Based and In-Person Education on Ill Health Self-management Skills in Adults With Bipolar Disorder: Protocol for a Mixed Methods Study” (JMIR Res Protoc 2021;10(9):e25168) one error was noted.

One author's name was displayed as:

Andreas Hatzittofis

It has now been corrected to:

Andreas Chatzittofis

The correction will appear in the online version of the paper on the JMIR Publications website on September 15, 2021, together with the publication of this correction notice. Because this was made after submission to PubMed, PubMed Central, and other full-text repositories, the corrected article has also been resubmitted to those repositories.

